# Effects of MSM on exercise-induced muscle and joint pain: a pilot study

**DOI:** 10.1186/1550-2783-12-S1-P8

**Published:** 2015-09-21

**Authors:** Eric D Withee, Kimberly M Tippens, Regina Dehen, Douglas Hanes

**Affiliations:** 1 Helfgott Research Institute, National College of Natural Medicine, Portland, OR 97201, USA

## Background

Participants in organized running commonly experience muscle and joint pain while training for and competing in distance events. Many runners report pain as a major influence on changes or breaks in training regimens, and as a common deterrent for returning to exercise after a break. Methylsulfonylmethane (MSM) is a sulfur-based nutritional supplement shown through several clinical trials to be effective in reducing pain associated with osteoarthritis, and to exhibit anti-inflammatory properties. To further investigate the role of MSM in pain management, this randomized, double-blind, placebo-controlled study evaluated the effects of MSM supplementation on exercise-induced muscle and joint pain.

## Methods

Twenty-two healthy females (n = 17) and males (n = 5) (33.7 ± 6.9 yrs.) were recruited from the 2014 Portland Half-Marathon registrant pool. Participants were randomized to take either MSM (OptiMSM^®^) (n = 11), or a placebo (n = 11) at 3g/day for 21 days prior to the race and two days after (23 total). Pain was recorded using a 100 mm Visual Analogue Scale (VAS) for both muscle pain (MP) and joint pain (JP) on a single questionnaire. Participants completed the questionnaire at five time points. Baseline levels (T_0_) were recorded approximately one month prior to the race. Post-race pain levels were recorded at 15 minutes (T_1_), 90 minutes (T_2_), 1 Day (T_3_), and 2 days (T_4_) after race finish. Data were analyzed using linear mixed models controlled for baseline, with time point as a repeated factor. Simple contrasts compared post-race time points to baseline, and Student's t-tests assessed between-group time point comparisons.

## Results

Half-marathon completion resulted in significant time effects for increased pain in both MP (p < 0.001) and JP (p < 0.001). Mean MP at T_0 _(14.7mm) significantly increased at T_1 _(38.4mm; p < 0.001), T_2 _(33.5mm; p = 0.001), and T_3 _(36.3mm; p = 0.001), and fell to non-significant levels at T_4 _(20.9mm; p = 0.330). Mean JP at T_0 _(8.4mm) significantly increased at T_1 _(33.5mm; p < 0.001), T_2 _(31.5mm; p < 0.001), and T_3 _(24.8mm; p = 0.004), and fell to non-significant levels at T_4 _(16.1 mm; p = 0.198). The results showed a trend of lower pain levels in the MSM group. However, time-by-treatment effects did not reach significance in either MP or JP. Compared to placebo, MSM supplementation resulted in nearly significantly lower MP at T_1 _(MSM = 27.3mm vs. placebo = 49.8mm, p = 0.063), and lower MP at T_2 _(27.1mm vs. 40.0mm; p = 0.300), and T_3 _(30.0mm vs. 41.9mm; p = 0.306). Similar results were seen for JP at T_1 _(24.2mm vs. 42.4mm; p = 0.156), T_2 _(22.7mm vs 39.3mm; p = 0.204), and T_3 _(15.4mm vs. 32.2mm; p = 0.152).

## Conclusion

Exercise-induced muscle pain and joint pain increase within 15 minutes of completing a half-marathon, continue through the following day, and diminish approximately two days post-race. Three weeks of MSM supplementation at 3g/day attenuated post-exercise muscle and joint pain at clinically significant levels compared to placebo. However, the pain reductions did not reach statistical significance, warranting further research on MSM and post-exercise pain among larger samples.

